# Emergence of Plasmodium vivax Resistance to Chloroquine in French Guiana

**DOI:** 10.1128/AAC.02116-18

**Published:** 2019-10-22

**Authors:** Lise Musset, Christophe Heugas, Richard Naldjinan, Denis Blanchet, Pascal Houze, Philippe Abboud, Béatrice Volney, Gaëlle Walter, Yassamine Lazrek, Loïc Epelboin, Stephane Pelleau, Pascal Ringwald, Eric Legrand, Magalie Demar, Félix Djossou

**Affiliations:** a Laboratoire de Parasitologie, Centre National de Référence du Paludisme, World Health Organization Collaborating Center for Surveillance of Anti-Malarial Drug Resistance, Institut Pasteur de la Guyane, Cayenne, French Guiana, France; b Faculty of Medicine, Université de Poitiers, Poitiers, France; c Department of Infectious and Tropical Diseases, Centre Hospitalier Andrée Rosemon, Cayenne, French Guiana, France; d Laboratoire Hospitalo-Universitaire de Parasitologie et Mycologie, Centre Hospitalier Andrée Rosemon, Cayenne, French Guiana, France; e Biochemistry Laboratory, Hôpital Saint Louis, Paris, France; f Global Malaria Programme, World Health Organization, Geneva, Switzerland; g Malaria Genetic and Resistance Group, Biology of Host-Parasite Interactions Unit, Institut Pasteur, Paris, France

**Keywords:** *P. vivax*, resistance, Amazonia, French Guiana, Guiana Shield, chloroquine, *pvcrt-o*, *pvmdr1*

## Abstract

In South America, Plasmodium vivax resistance to chloroquine was recently reported in Brazil and Bolivia. The objective of this study was to collect data on chloroquine resistance in French Guiana by associating a retrospective evaluation of therapeutic efficacy with an analysis of recurrent parasitemia from any patients. Patients with P. vivax infection, confirmed by microscopy and a body temperature of ≥37.5°C, were retrospectively identified at Cayenne Hospital between 2009 and 2015. Follow-up and treatment responses were performed according to the World Health Organization protocol. Parasite resistance was confirmed after dosage of a plasma concentration of chloroquine and microsatellite characterization. The *pvmdr1* and *pvcrt-o* genes were analyzed for sequence and gene copy number variation. Among the 172 patients followed for 28 days, 164 presented adequate clinical and parasitological responses. Eight cases of treatment failures were identified (4.7%; *n* = 8/172), all after 14 days. The therapeutic efficacy of chloroquine was estimated at 95.3% (95% confidence interval [CI], 92.5 to 98.1%; *n* = 164/172). Among the eight failures, five were characterized: two cases were true P. vivax chloroquine resistance (1.2%; 95% CI, 0 to 2.6%; *n* = 2/172), and three cases were found with subtherapeutic concentrations of chloroquine. No particular polymorphism in the Plasmodium vivax *pvmdr1* and *pvcrt-o* genes was identified in the resistant parasites. This identified level of resistance of P. vivax to chloroquine in French Guiana does not require a change in therapeutic recommendations. However, primaquine should be administered more frequently to limit the spread of resistance, and there is still a need for a reliable molecular marker to facilitate the monitoring of *P. vivax* resistance to chloroquine.

## Abstract (precis)

In South America, Plasmodium vivax resistance to chloroquine was recently reported in Brazil and Bolivia. The objective of this study was to collect data on chloroquine resistance in French Guiana by associating a retrospective evaluation of therapeutic efficacy with an analysis of recurrent parasitemia from any patients. Patients with P. vivax infection, confirmed by microscopy and a body temperature of ≥37.5°C, were retrospectively identified at Cayenne Hospital between 2009 and 2015.

## INTRODUCTION

In 2017, malaria was still the most prevalent parasitic disease in the world, with 1.4 billion people remaining at risk (1). Representing 40% of malaria cases worldwide, Plasmodium vivax was the second species most responsible for human malaria after Plasmodium falciparum and is the most frequent species outside Africa. The same year in French Guiana, an overseas French territory located on the Guiana Shield in South America, 86% of malaria cases were due to P. vivax, and the remaining were due to P. falciparum, with scarce reports of Plasmodium malariae cases. In this region, the incidence of P. vivax exceeded that of P. falciparum in 2005 (2, 3). Meanwhile the overall number of notified malaria cases decreased from 4,000 in 2009 to 597 in 2017 (4).

Since 1995 in French Guiana, chloroquine (CQ) has no longer been recommended for treatment of P. falciparum and has been replaced by quinine-doxycycline before artemether-lumefantrine (3). However, it is the standard treatment for uncomplicated P. vivax infection. Its posology follows the World Health Organization (WHO) recommendation: an oral dose of 25 mg/kg of body weight distributed over 3 days and 14 days of 30-mg/day primaquine (PQ) to cure dormant hypnozoites (3, 5). Unfortunately, PQ is not systematically administered, mainly because of administrative constraints and difficulties in assessing the glucose-6-phosphate dehydrogenase (G6PD) activity of P. vivax malaria patients in remote areas (3). Without appropriate treatment, these dormant liver forms can cause relapses and participate in transmission (6). As relapses may be caused by the homologous or heterologous genotype, the genetic characterization of parasites is not very useful to characterize failures (7), thus limiting the study of antimalarial drug effectiveness against P. vivax.

P. vivax multiplication should not occur within 35 days after an adequate CQ treatment. During this time, the mean whole-blood concentrations of CQ and its metabolite desethylchloroquine (dCQ) are normally greater than 100 ng/ml and prevent parasite multiplication. CQ resistance (CQR) is suspected if parasitemia increases during this period (8). Therefore, P. vivax resistance could be identified using plasma concentrations (9). *In vitro* phenotyping methods are scarce and difficult to implement especially because of the very low synchronicity of parasites belonging to the Chesson South American strain (10). Putative molecular markers of CQR have been identified by homology with those from P. falciparum. Positions 976 and 1076 of the P. vivax multidrug resistance 1 gene (*pvmdr1*) have been associated with resistance without clear evidence (11, 12). *pvmdr1* is considered only a minor determinant for resistance to chloroquine, eventually considered more informative for resistance to mefloquine (13, 14). *pvcrt-o*, the ortholog gene of *pfcrt*, the P. falciparum molecular marker for resistance to several antimalarial drugs, has also been described as a putative marker for resistance of P. vivax to chloroquine (15, 16). Gene duplication and the expression level of these genes have also been described as genetic determinants (17).

The first descriptions of well-documented P. vivax resistance in the Americas came from the Republic of Guyana, also part of the Guiana Shield (18). More recently in Oiapoque, Brazil, on the border with French Guiana, 1.1% (*n* = 1/95) of treatment failures (TFs [i.e., recurrent parasites after treatment]) were reported after supervised treatment with the combination CQ+PQ (19). Manaus, Amazonas, Brazil, is nowadays the hot spot of P. vivax resistance in South America, with 10.1% of TFs (*n* = 11/109) reported after supervised CQ treatment (20) or 5.2% (*n* = 7/135) after concomitant administration of CQ+PQ (21).

The present study’s main objective was to bring out new data on P. vivax CQR in French Guiana. This combined study associated the results from a follow-up therapeutic efficacy study of CQ implemented in clinical practices at the Cayenne Hospital with a retrospective analysis of recurrence in patients presenting fever and positive parasitemia within 35 days after the initial infection.

## RESULTS AND DISCUSSION

### In Cayenne Hospital, the patients infected by P. vivax are mostly young men.

**FIG 1:**
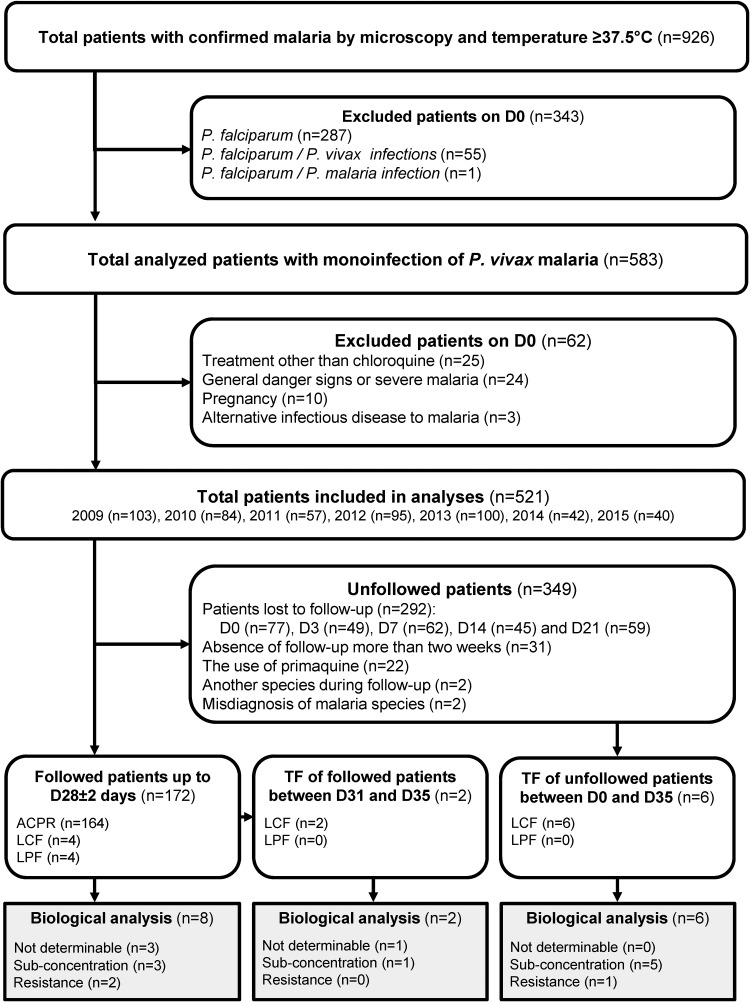
Malaria infections diagnosed at Cayenne Hospital, French Guiana, 2009 to 2015. ACPR, adequate clinical and parasitological response; D0 to D35, days 0 to 35; LCF, late clinical response; LPF, late parasitological response.

Between 2009 and 2015, 926 patients were screened and diagnosed positive for malaria (Fig. 1). During the clinical examination of the 583 patients with P. vivax infections, the median body temperature was 39.9°C (range, 37.5 to 41.5°C). The median parasitemia was 0.57% (range, 0.01 to 2.00%). During this period, young men were mostly infected by P. vivax (male/female [M/F] sex ratio, 2.05; median age, 31 years [range, 1 to 76 years]). Patients were diagnosed a median of 3 days after their first symptoms. No patient was underweight, and 22.5% (*n* = 131) of them declared vomiting.

### Therapeutic efficacy evaluated in one-third of the patients based on the standard hospital protocol for malaria care.

Of the 583 enrolled patients with P. vivax infections, 62 were excluded from the analysis on day 0 (D0) (Fig. 1). Of the 521 remaining patients, 172 patients were followed up to D28, and 292 were lost during follow-up. Fifty-seven patients were excluded during the study (Fig. 1). Among those 172 patients, men were more affected by malaria than women (M/F sex ratio, 2.37) (Table 1). The median age was 32 years (range, 5 to 76 years). Only 13.4% (*n* = 18) of these 172 patients took a chemoprophylaxis treatment: mainly (94.4%) members of the military using doxycycline. A history of malaria within the last 3 months was reported for 37.6% (*n* = 56/149) of patients. Within the same period, a history of travelling out of Cayenne was recorded for the majority of patients, of which 71.2% (*n* = 104/146) declared they had traveled within French Guiana, while 28.8% (*n* = 42/146) reported not having traveled. Cayenne Hospital is located outside the malaria transmission areas in French Guiana. However, it concentrates people from all over French Guiana, including people contaminated in gold mines (at least *n* = 32 [data not shown]), such as gold miners or members of the military dedicated to fighting illegal gold mining activities. Thus, the analyzed sample set of this study could be considered representative of the general parasite population circulating in the country.

**TABLE 1:** Patient characteristics of uncomplicated P. vivax malaria treated by 3 days of chloroquine administration in French Guiana from 2009 to 2015^*a*^

Parameter	Result for:			*P*
	Total	ACPR on D28	Recurrent parasitemia on D28	
Patients, no. (%)				
Total	172	164	8	
By yr				0.554
2009	38 (22.1)	37 (97.4)	1 (2.6)	
2010	37 (21.5)	35 (94.6)	2 (5.4)	
2011	24 (14.0)	24 (100.0)	0 (0.0)	
2012	44 (25.6)	41 (93.2)	3 (6.8)	
2013	11 (6.4)	10 (90.9)	1 (9.1)	
2014	8 (4.6)	7 (87.5)	1 (12.5)	
2015	10 (5.8)	10 (100.0)	0 (0.0)	
Gender, no. (%)				0.768
M/F ratio	2.37	2.35	3.00	
Male	121 (70.4)	115 (95.0)	6 (5.0)	
Female	51 (29.7)	49 (96.1)	2 (3.9)	
Age, median yr (range)	33.00 (5–76)	33.00 (5–76)	30.50 (17–56)	0.134
Age group, no. (%)				0.496
Adults	163 (94.8)	155 (95.1)	8 (4.9)	
5–15 yr old	9 (5.2)	9 (100.0)	0 (0.0)	
History of malaria, no. (%)				<0.001*
Yes	56 (32.5)	55 (98.2)	1 (1.8)	
No	93 (54.1)	91 (97.8)	2 (2.2)	
Unknown	23 (13.4)	18 (78.3)	5 (21.7)	
Prophylaxis, no. (%)				0.121
Yes	18 (10.5)	18 (100.0)	0 (0.0)	
No	116 (67.4)	112 (96.6)	4 (3.4)	
Unknown	38 (22.1)	34 (89.5)	4 (10.5)	
Days before consultation, median no. (range)	3 (0–15)	3 (0–15)	1 (0–7)	0.428
Body wt, median kg (range)	72 (49–158)	72 (49–158)	68 (58–83)	0.493
Body temp, median °C (range)	39.0 (37.7–40.9)	39.0 (37.7–40.9)	38.9 (37.5–39.6)	0.672
Vomiting, no. (%)				0.671
Yes	59 (34.3)	57 (96.6)	2 (3.4)	
No	106 (61.6)	100 (94.3)	6 (5.7)	
Unknown	7 (4.1)	7 (100.0)	0 (0.0)	
Parasitemia, median % infected blood cells (range)	0.15 (0.01–2.00)	0.15 (0.01–2.00)	0.13 (0.02–0.76)	0.218
Hospitalization, no. (%)				0.946
Yes	23 (13.4)	22 (95.7)	1 (4.3)	
No	98 (57.0)	93 (94.9)	5 (5.1)	
Unknown	51 (29.6)	49 (96.1)	2 (3.9)	

a ACPR, adequate clinical and parasitological response; D28, day 28. *, *P* < 0.05 (significant difference).

### High but incomplete (95.3%) CQ therapeutic efficacy against P. vivax in French Guiana between 2009 and 2015.

In French Guiana, between 2009 and 2015, the D28 follow-up estimated a therapeutic efficacy of CQ at 95.3% (95% confidence interval [CI], 92.5 to 98.1; *n* = 164/172) to treat uncomplicated P. vivax monoinfection. This study included a large number of patients, compared to the WHO recommendation (*n* = 172 versus 73). Therefore, these results were associated with a confidence level of 95% and a margin error of 2.8%. A cross-analysis was done within samples received at the National Reference Center (around 50% of the total number declared each year in the country) to track potential recurrent parasitemia in patients enrolled in the protocol but followed outside the Cayenne Hospital. This allowed us to identify one additional recurrent parasitemia. This confirms the robustness of the results presented from this Cayenne Hospital follow-up. However, recurrent cases could be underestimated in miners as they rarely complete the follow-up regardless of the medical recommendations so they can rapidly travel back into the deep forest (22).

With an endpoint at D28, eight patients experienced recurrent parasitemia (4 with late clinical failure [LCF] and 4 with late parasitological failure [LPF])—all after D14. No difference between years of infection was observed (*P* = 0.5542 [Table 1]). Therapeutic efficacy was stable for a 7-year period, suggesting that drug pressure on the parasite population did not participate in a rapid spread of resistance through the parasite population. The only significant difference between the adequate clinical and parasitological response (ACPR) group and the group experiencing recurrent parasitemia was history of malaria (*P* = 0.0022). Therefore, in the absence of systematic PQ prescription, this observation could suggest a large part of recurrence was due to relapses.

### P. vivax resistance occurs at the minimum prevalence of 1.2%.

**FIG 2:**
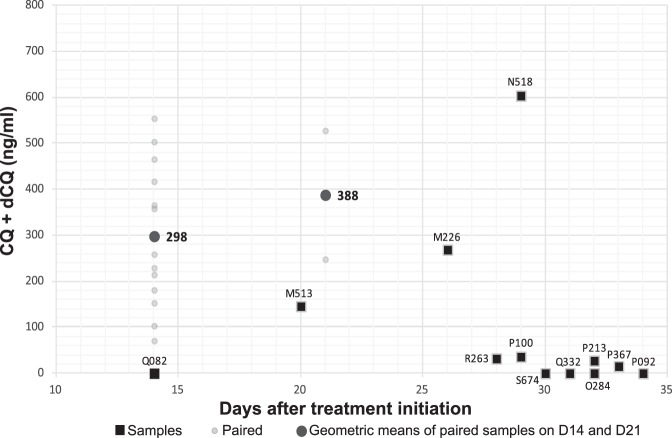
Chloroquine plasma concentrations (including chloroquine [CQ] plus desethylchloroquine [dCQ]) per day in 16 patients experiencing treatment failure (black squares) and 15 patients with adequate treatment responses (small gray circles) in French Guiana from 2009 to 2015. Mean of adequate treatement response concentrations are also represented (large gray circles).

Two out of five analyzable failures on the D28 follow-up had a drug level that normally kills or at least suppresses parasite multiplication (M513, 146 ng/ml on D20; N518, 604 ng/ml on D29 [Table 2]). After comparison of plasma dosages of CQ with those of samples associated with ACPR around the same day of follow-up, these concentrations were compatible with an efficient antimalarial activity on sensitive parasites (Fig. 2). These results demonstrated that parasite resistance to CQ was present in at least 1.2% (95% CI, 0 to 2.6%; *n* = 2/172) of the patients in French Guiana. The six microsatellite markers showed homologous genetic background of parasites at D0 and the day of treatment failure in these cases (DF).

**TABLE 2:** Characterization of P. vivax samples associated with a recurrent parasitemia after chloroquine treatment, French Guiana, 2009 to 2015^*a*^

ID	BT (°C)	Parasitemia (%)	Treatment	Treatment response on D28	CQ+dCQ (ng/ml)	*Pvmdr1* copy no.	*Pvmdr1* sequence	*Pvcrt-o* copy no.	Insertion of *pvcrt-o* K10	Sizes of microsatellite loci 13.239, 3.27, 5.504, 11.162, MS9, and MS8^*f*^
Recurrence in patients followed D28 ± 2 days										
Not determinable reasons^*b*^										
P063										
D0	39.0	0.1000	CQ			ND	A/WT	0.94 ± 0.19	Yes	192–196, 109–117, 206–213–272, 182–186, 112–120, 199–207–211
D22	36.1	0.1000	No	LPF	NR	ND	A	ND	ND	192–196, 109, ND, 182–186, 112–120, 199–207
N183										
D0	39.0	0.1800	CQ			NR	NR	NR	NR	NR
D30	38.7	0.3000	CQ	LCF	NR	NR	NR	NR	NR	NR
NR										
D0	38.9	0.1000	CQ			NR	NR	NR	NR	NR
D22	39.0	1.2000	CQ	LCF	NR	NR	NR	NR	NR	NR
Subtherapeutic chloroquine concentrations observed^*c*^										
P100										
D0	39.6	0.1600	CQ			2	A	0.76 ± 0.02	No	192, 133, 199, 262–266–270, 116, 203
D29	36.8	0.0010	No	LPF	36 + <10	2	A	0.61 ± 0.01	No	Identical to D0
Q082										
D0	38.1	0.0300	CQ			1	A	0.79 ± 0.05	Yes	191–196, 109, 59, 182, 112–116–120, 207
D14	37.0	0.0300	No	LPF	<10 + <10	1	A	0.73 ± 0.01	Yes	Identical to D0
R263										
D0	36.0	0.0020	CQ			1	A	0.69 ± 0.07	Yes	192, 109, 213, 186, 116-120-124, 199
D28	39.0	0.1400	CQ	LCF	17 + 15	1	A	0.97 ± 0.01	Yes	Identical to D0
Chloroquine therapeutic failure associated with parasite resistance^*d*^										
M513										
D0	38.9	0.2500	CQ			1	A	1.01 ± 0.08	Yes	200, 129, 213, 186, 108, 207
D2	36.7	0.0005								
D6	36.5	0.0000								
D13	37.6	0.0000								
D20	36.5	0.0000			93 + 53					
D26	36.7	0.0005	No	LPF	NR	1	A	ND	ND	Identical to D0
N518										
D0	38.8	0.7600	CQ			1	A	1.09 ± 0.04	No	192, 109, 213, 186, 116–120, 207
D1	NR	0.0000								
D3	NR	0.0000								
D13	NR	0.0000								
D29	39.5	0.2000	CQ	LCF	224 + 379	1	A	0.78 ± 0.03	No	Identical to D0
Extended or no follow-up^*e*^										
Not determinable reasons^*b*^										
NR										
D0	39.2	1.0000	CQ			NR	NR	NR	NR	NR
D35	40.0	0.6000	CQ	ACPR	NR	NR	NR	NR	NR	NR
Subtherapeutic chloroquine concentrations observed^*c*^										
O284										
D0	39.2	0.5000	CQ			1	A/WT	1.48 ± 0.04	No	192, 109–129-149, 213, 186, 116–120-124, 207
D32	38.6	0.4500	CQ	Lost	<10 + <10	1	A/WT	0.96 ± 0.15	No	Identical to D0
P092										
D0	39.0	0.4600	CQ			1	A	0.83 ± 0.09	No	188, 109, 213, 186, 116–120–124, 199
D34	39.7	0.1700	CQ	Lost	<10 + <10	2	A	1.33 ± 0.21	Yes	192, 109, 213, 186, 116-120-124, 199
P213										
D0	39.0	0.1000	CQ			2	A	0.80 ± 0.06	No	196, 121, 262, 186, 116–120–124, 215-219
D32	39.0	0.0700	CQ	ACPR	17 + 10	2	A	0.70 ± 0.08	No	Identical to D0
P367										
D0	38.3	0.1000	CQ			1	A	0.75 ± 0.03	Yes	196, 109, 213, 182, 116-120, 207
D33	38.6	0.1100	CQ	Lost	15 + <10	1	A	1.19 ± 0.15	Yes	Identical to D0
Q332										
D0	37.6	0.3500	CQ			1	A	1.11 ± 0.03	No	192–196, 97–145, 206–213, 182–186, 112–116–120, 207
D31	40.0	0.0500	CQ	Lost	<10 + <10	1	A	1.00 ± 0.04	No	Identical to D0
S674										
D0	39.5	0.1800	CQ			1	A	1.02 ± 0.02	No	192, 129, 59-206, 182, 124–128–132, 199
D30	38.1	0.0200	CQ	Lost	<10 + <10	1	A	0.74 ± 0.13	No	Identical to D0
Chloroquine therapeutic failure associated with parasite resistance^*d*^										
M226										
D0	38.6	0.2000	CQ			2	A	0.89 ± 0.02	Yes	196, 109, 213, 186, 120–124, 219
D7		0.0000								
D26	38.6	0.0500	CQ	Lost	65 + 204	2	A	0.64 ± 0.06	Yes	Identical to D0

a A, Guy-A; ACPR, adequate clinical and parasitological response; BT, body temperature; D0 to D35, days 0 to 35; dCQ, desethylchloroquine; ID, identification; LCF, late clinical response; Lost, lost to follow-up; LPF, late parasitological response; ND, not determinable; No, no modification of the treatment; NR, not received; *pvmdr1*, Plasmodium vivax multidrug resistance 1 gene; WT, wild type. *Pvcrt-o* gene copy numbers have been determined based on two technical replicates.

b Reasons for recurrence not determinable because no drug concentration was available.

c Recurrence linked to subtherapeutic concentration of drug: the CQ+dCQ concentration was <100 ng/ml.

d Treatment failure (i.e., parasite resistance): the CQ+dCQ concentration was >100 ng/ml, with the same parasite genotype observed on D0 and DF.

e Recurrence observed in patients with extended follow-up (D31 to D35) or patients without follow-up (D1 to D35).

f Values separated with a hyphen mean that this microsatellite has a multiclonal stucture represented by the different observed sizes at the studied locus.

Until 1995, chloroquine was also recommended to treat P. falciparum (3). However, resistance of P. vivax has not evolved as quickly as for P. falciparum. French Guiana is the second country of the Guiana Shield reporting cases of CQR to P. vivax after the Republic of Guyana. In South America, resistance was reported in Manaus, Brazil (20), in more than 10% of cases after CQ treatment. Other studies also reported CQR in Amazonia but after CQ+PQ treatment: 5.2% in Manaus, Brazil (CQ+PQ) (21), 1.1% in Oiapoque, Brazil (CQ+PQ) (19), and 6.5% in Bolivia (CQ+PQ) (23). However, comparison with these results is impossible because of the potentialization of CQ action by PQ when the drugs are coadministered (24). In France, coadministration is rare because PQ is never given without a preliminary evaluation of the G6PD activity of the patient by quantitative laboratory methods.

Beside the eight treatment failures observed during the D28 follow-up, eight cases of treatment failures were also identified from the D35 extended follow-up or patients who were not followed but had returned to the hospital because of fever (Fig. 1). Biological analyses were conducted to identify resistance in seven of these samples because for one case, plasma and/or DNA was missing. In this context, one new case of P. vivax resistance was identified with a CQ+dCQ plasma concentration ([CQ]) of 269 ng/ml (Fig. 2). The other six were associated with very low or undetectable chloroquine concentration despite the fact that until day 35, the chloroquine concentration should normally be above 100 ng/ml (8). Therefore, these recurrent cases of parasitemia were probably due to relapses. In fact, the Chesson strain circulating in South America generates relapses around D28 (25). With a limited and variable [CQ] in blood after D28, these results underlined the importance of ending the follow-up at D28 during a chloroquine efficacy study in case of infection by a Chesson strain, before the occurrence of natural relapses.

### A relevant molecular marker is required to easily monitor *P. vivax* resistance to chloroquine.

**FIG 3:**
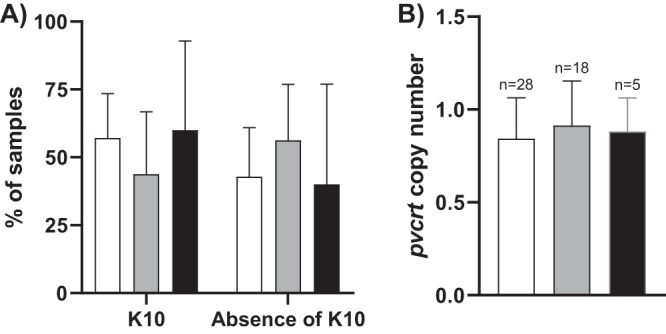
Genotyping of the *pvcrt-o* gene. (A) Prevalence of the insertion of K10. (B) Gene copy number. Results are presented according to the therapeutic response to chloroquine for each group of P. vivax isolates: adequate clinical and therapeutic response in white (*n* = 28), failure in the absence of resistance in gray (*n* = 9 D0-DF pairs), and failure associated with parasite resistance in black (M226 and N518 D0-DF pairs and M513 D0).

The genotype and copy number of the *pvmdr1* and *pvcrt-o* genes were analyzed in the 13 available pairs of samples (D0-DF) associated with a treatment failure whatever the reason. The *pvmdr1* mutation T958M previously described as prevalent in French Guiana was identified in 86.5% of the samples (26). No difference within D0-DF pairs of samples was observed. The mutations Y976F and F1076L were absent even in the chloroquine-resistant parasites associated with the three chloroquine treatment failures (M226, M513, and N518). The *pvmdr1* copy number was 2 in seven samples (29.2%), without any associated with the *in vivo* phenotype. The general percentage of multicopy samples was significantly higher than what was observed in the same period in the general parasite population of French Guiana (12.8%; *n* = 43/335; *P* < 0.05) (data not shown). The *pvcrt-o* part of the gene including an extra amino acid at position 10 (K10) has been sequenced in D0-DF pairs as well as in a sample set of 28 samples in order to compare to the genetic profile specific of French Guiana (27). No difference within D0-DF pairs of samples was observed for the *pvcrt-o* K10 insertion (Table 2). This polymorphism was observed in 57.1% of samples (95% CI, 38.8 to 75.5%; *n* = 16/28 [Fig. 3A]). The *pvcrt-o* gene was monocopy in the sample set, whether associated or not with therapeutic failure (Fig. 3B).

The *pvmdr1* and *pvcrt-o* genotypes (sequence and copy number) were not associated with CQR in French Guiana. As previously described, these markers are probably not markers of chloroquine resistance or even minor determinants for resistance (13). However, their expression levels have been described as being associated with P. vivax resistance in the Amazon region (17). These have not been analyzed in this study because of the absence of RNA collection.

### P. vivax resistance to CQ exists in French Guiana and needs to be controlled using primaquine.

P. vivax CQR exists in French Guiana but at a low prevalence. Therefore, these results do not justify a change in treatment regimen and regional recommendations. However, a better implementation of the coadministration of CQ and PQ is crucial to avoid the spread of these resistant parasites. To do so, the use of rapid quantitative screening methods for G6PD deficiency and the systematic recording of patient’s G6PD status should be considered.

## MATERIALS AND METHODS

### Study site, patients, and treatment monitoring.

All cases of malaria confirmed by microscopy and an axillary temperature of ≥37.5°C or subjects with a history of fever during the past 24 h between March 2009 and October 2015 were retrospectively included in this study. Cases were excluded from the analysis under the following circumstances: (i) cases of severe P. vivax malaria according to the WHO definitions regarding P. falciparum, (ii) the presence of concomitant infectious disease or comorbid conditions, (iii) pregnant women, and (iv) patients not treated by CQ (Nivaquine) at a total dose of 25-mg base/kg body wt (10-mg base/kg body wt on day 0 [D0], 10 mg/kg body wt on D1, and 5 mg/kg body wt on D2). Treatment administration was not supervised.

Patients treated by antimalarial drugs followed the common clinical practices of Cayenne Hospital. Patients were invited to come back for clinical and biological examinations on D3, D7 ± 1, D14 ± 1, D21 ± 2, and D28 ± 2. Hospitalized patients were also followed on D1 and D2. Patients who did not experience TF during the standard 28-day follow-up had an extended follow-up until D35. Additionally, patients were informed to come back to the hospital in case of symptom resurgence without waiting for the next scheduled visit.

Any patients who did not attend the visit on D28 were classified as lost to follow-up. However, patients who attended the D28 visit but were not followed for more than 2 weeks during this period were withdrawn from the analysis. Those who missed one appointment but had no relapse detected during the preceding and following appointments were considered to have negative parasitemia for the missed appointment and were not withdrawn from the study. Finally, patients (i) treated with PQ, (ii) misdiagnosed on D0, or (iii) diagnosed with another malaria species during the follow-up were also excluded.

General baseline data were also recorded in the patients’ files: (i) sex, (ii) age, (iii) history of malaria in the last 3 months, (iv) onset of symptoms, (v) weight, (vi) history of travel during the 4 weeks preceding the consultation, and (vii) antimalarial chemoprophylaxis.

### Classification of treatment responses.

Treatment responses were classified according to the WHO guidelines (5), as early treatment failure (ETF), late clinical failure (LCF), late parasitological failure (LPF), or adequate clinical and parasitological response (ACPR). Treatment failures (TFs) included ETF, LCF, and LPF.

In order to properly characterize parasite resistance, biological analyses were conducted on samples from all patients who had experienced recurrent parasitemia, including (i) patients presenting TF during the standard or extended follow-up and (ii) patients not followed but diagnosed with TF outside the protocol.

### Measurement of antimalarial drug concentration.

CQ+dCQ plasma concentrations ([CQ]) were measured at the day of treatment failure (DF) by liquid chromatography combined with tandem mass spectrometry (TSQ Quantum Ultra; Thermo Fisher, France) as previously reported by Hodel et al., with minor modifications (28). Using OASE 96-well microplates (Waters, France), 100 μl of plasma was mixed with acetonitrile. Proteins and phospholipids were eliminated by positive pressure using the 96-Positive Pressure system (Waters, France). Eluents were evaporated at room temperature. Dry residues were dissolved in 100 μl of mobile phase, and 10 μl was injected into the system. For both molecules, the method was linear between 10 and 1,000 ng/ml. For homemade and external controls from the WorldWide Antimalarial Resistance Network, coefficients of variation were below 10% and bias values were ±10%.

A plasma concentration greater than 100 ng/ml was considered adequate up to D35 (8). When the measurement was conducted before D28, results were compared to drug concentrations observed in patients with adequate clinical and parasitological responses on the same day of follow-up. Then TFs were classified as (i) TF due to subtherapeutic concentration if [CQ] is <100 ng/ml, (ii) TF due to resistance if [CQ] is >100ng/ml, and (iii) “unclassified” if no drug concentration was available.

### DNA extraction.

Parasite DNA was extracted from 200 μl of blood using QIAamp genomic DNA kits according to the manufacturer’s instructions (Qiagen, Courtaboeuf, France).

### Microsatellite characterization.

The genetic background of parasites was compared between D0 and DF using a set of six microsatellite loci (3.27, 8.504, 11.162, 13.239, MS8, and MS9) (29–31). This panel was selected because of its high polymorphism in the general parasite population of French Guiana (0.62 < expected heterozygosity [*H*_E_] < 0.68 [data not shown]). Microsatellites were analyzed by nested PCR following previously described procedures and with the primers listed in Table S1 in the supplemental material. The homologous genetic profile (based on the allelic sizes) between D0 and DF suggested a recrudescence of resistant forms, while heterogeneous profiles suggested a new infection. However, whatever the genetic profile, relapses could not be excluded.

### Analysis of *pvmdr1* and *pvcrt-o* as putative molecular markers of CQR.

The *pvmdr1* and *pvcrt-o* gene sequences and gene copy numbers were analyzed on all isolates associated with a recurrence collected on D0 and DF according to the previously described methods (26, 27). PCR products were visualized using 2% agarose gel electrophoresis before a double-strand Sanger sequencing. The generally accepted Sanger sequencing limit in case of a mixed genotype is around 10% for the minor genotype (32). Sequences were analyzed with Geneious 8.1.7 software (Biomatters, Ltd., Auckland, New Zealand). Positive and negative controls were systematically included in each series of genotyping. In the absence of known published genotype for the *pvcrt-o* gene, a sample set of 28 samples associated with adequate treatment response has also been analyzed in order to compare the results.

### Ethical and consent approval.

Data and samples were all obtained as standard medical care for any patient presenting fever on hospital admission in French Guiana. According to the French legislation (article L.1211-2 of the French Public Health Code), biobanking and secondary use for scientific purpose of data and human clinical samples are possible as long as the corresponding patients are informed and have not given any objection. In our study, information was given to every patient through the Cayenne Hospital brochure, and no immediate or delayed patient opposition was reported. In cases involving infants, parents or guardians had to report their opposition to the hospital. Samples received from the National Reference Center (NRC) biobank were approved and registered by the French Ministry for Research and the French Ethics Committee (declaration no. DC-2010-1223, collection Nu2). According to the French legislation, no institutional review board approval was required.

### Statistical analysis.

Data were collected with Microsoft Excel 2016 (Microsoft, Redmond, WA, USA). Statistical computing was analyzed with R software (R Foundation, Vienna, Austria). Percentages were calculated according to the total number of patients followed up to D28 with a 95% confidence interval. Medians were associated with range. The Wilcoxon test and Fisher’s test were performed to compare data between ACPR and TF after 28 ± 2 days of follow-up. A *P* value of <0.05 was considered significant.

## Supplementary Material

Supplemental file 1
