# Supplementary material for: Emergence of Plasmodium vivax Resistance to Chloroquine in French Guiana
Source: Antimicrob Agents Chemother. 2019 Oct 22;63(11):e02116-18. doi: 10.1128/AAC.02116-18 (PMC6811453; doi:10.1128/AAC.02116-18)
Supplement: Supplemental file 1 [file AAC.02116-18-s0001.pdf]

SUPPORTING INFORMATION

S1 Table : Microsatellite characterization of *P. vivax* infections

	Primary PCR				Nested PCR			
**Markers**	**Primers 5' → 3'**	**Hybridization (°C)**	**[MgCl2] (mM)**	**Cycle number**	**Primers 5' → 3'**	**Hybridization (°C)**	**[MgCl2] (mM)**	**Cycle number**
**3.27 (locus1)**	A180 : F-GCGATTAAGCTGCACTGAAT	60.0	2	20	A181 : F-D3*-ACCCTCCAATGAGCACAAG	60.0	2	25
	A182 : R-TCCAAATGTATGTGCAGTCG				A182 : R-TCCAAATGTATGTGCAGTCG			
**8.504 (locus6)**	A171 : F-TTCTTTCATAGGAATAGCTGC	55.0	2	20	A172 : F-D3*-TCTTCTCGTCTCCTTTTCTG	60.0	2	25
	A173 : R-AGTGTGTGTAGTGGGTGGAG				A173 : R-AGTGTGTGTAGTGGGTGGAG			
**11.162 (locus5)**	A174 : F-GTAGGAACACGCCACGTT	60.0	2	20	A175 : F-D4*-TTTGTTAGGAGATCCGTCTG	60.0	3	25
	A176 : R-TTAAATGACACTTTGGCTTCC				A176 : R-TTAAATGACACTTTGGCTTCC			
**13.239 (locus2)**	A177 : F-CAACAACAAAATGAATGACG	57.5	2	20	A177 : F-CAACAACAAAATGAATGACG	55.0	3	25
	A178 : R-ATATGCCCATTCTGCAAGT				A179 : R-D2*-CCTACCTTTACGCGTTT			
**pvMS8 (locus3)**	A159 : F-TCCGTTGTTTTGTTGCCC	61.0	3	20	A159 : F-TCCGTTGTTTTGTTGCCC	55.0	1	25
	A160 : R-CACTTGTTCGTTCCGCTC				A161 : R-D4*-GCTAACCGATCGACCCAA			
**pvMS9 (locus4)**	A162 : F-TGTGGATAAGGGGAAAAA	53.6	3	20	A163 : F-D3*-AAACGTGCGTGATAGGT	52.3	5	40
	A164 : R-TTTTTCCTTGAGTTTACG				A164 : R-TTTTTCCTTGAGTTTACG			

Denaturation and elongation temperatures were respectively 94.0°C and 72.0°C for both primary and nested PCR.
